# Cold shock response in healthy children: reassessment and first comparison between cold and warm water immersion

**DOI:** 10.3389/fspor.2025.1610144

**Published:** 2026-01-12

**Authors:** S. Peter, A. Michaelis, R. Wagner, R. P. Marshall, M. Bovet, J. Weickmann, M. Weidenbach, I. Dähnert, C. Paech

**Affiliations:** 1 Department for Pediatric Cardiology, University of Leipzig—Heart Center, Leipzig, Germany; 2 Pediatric Practice, Leipzig, Germany; 3 RasenBallsport Leipzig GmbH, Leipzig, Germany; 4 Department of Orthopedic and Trauma Surgery, Martin-Luther-University Halle-Wittenberg, Halle, Germany

**Keywords:** children, cold shock response, diving, drowning, hyperventilation, immersion, swimming

## Abstract

**Introduction:**

Swimming and diving are popular recreational activities and essential skills to prevent death from drowning. While most drownings occur in cold water, cold shock response is discussed as a major cause of drowning. Until now, the data on the physiology of drowning and cold shock response in children are scarce, while drowning remains a significant concern in this population. This study was conducted to investigate the cold shock response in healthy children and compare cold and warm water immersion.

**Methods:**

Participants were first immersed up to the neck in warm water (34 °C, close to thermoneutral) and then in cold water (11 °C), while skin temperature, ECG, heart rate, respiratory rate, oxygen saturation and peripheral perfusion index were continuously monitored.

**Results:**

Heart rate and respiratory rate remained constant in warm water. In cold water, heart rate increased by 31% and respiratory rate by 58%, peaking at 30 s and beginning to normalize after 60 s.

**Conclusion:**

The current study presents new data on the cold shock response in healthy children and the first comparison between cold water immersion and warm water immersion in this population. Data showed that immersion into 11 °C (52 °F) cold water leads to significant increases in heart rate and respiratory rate, in contrast to immersion in warm water. Remarkably, there is a lower intensity of the cold shock response in children compared to adults.

## Introduction

Swimming and diving are popular recreational activities promoting an active lifestyle, enhance wellbeing, reduce obesity and lower cardiovascular risk (1, 2). Lack of these skills, particularly in children, limits social participation and increases drowning risk. Understanding the physiological responses to immersion and submersion and the physiology of drowning is essential to prevent death from drowning. Drowning is the third leading cause of unintentional injury death worldwide, accounting for about 7% of injury-related deaths, with the highest rates in children aged 1–9 (3). Physiologically, drowning involves two phases: immersion, triggering cold shock and hypothermia, and submersion, causing the diving response, autonomic conflicts, fear and water aspiration. The diving response refers to the parasympathetic response, including bradycardia, which is triggered by submersion, particularly facial immersion and apnea (4). Most drownings occur in water below body temperature, indicating that physiological responses are linked to cooling, involving skin cooling (cold shock), nerve and muscle cooling and core cooling (hypothermia). While hypothermia develops after ∼30 min in adults (4–7), children likely cool faster due to their higher surface-area-to-body-mass ratio. Bird et al. observed an average decrease in core body temperature of 2.5 °C per hour in children swimming in 15 °C water (8).

The cold shock response is a reaction of the sympathetic nervous system triggered by skin cold receptors in response to a sudden drop in temperature. It starts at a water temperature lower than 25 °C with a peak around 10–15 °C and lasts for 2–3 min, peaking at around 30 s, as reported in the adult population (5, 9). It induces tachycardia, hyperventilation and vasoconstriction (4). Moreover, hypoxia and hypercapnia resulting from an increased metabolic rate lead to an earlier attainment of the threshold for terminating breath-holding (10), while the respiratory center is directly stimulated, intensifying the respiratory drive (11). The maximum breath-hold time is decreased to a few seconds in water colder than 15 °C compared to 60–90 s at comfortable air temperature (4). Uncontrollable hyperventilation and reduced breath-hold time increase the risk of water aspiration, making cold shock a major drowning risk even for trained swimmers (4, 5, 12).. Furthermore, the occurrence of cardiac arrhythmias rises from 2% in cold water with free breathing to 82% when the face is immersed and breath is held. This may be caused by autonomic conflict from simultaneous activation of both branches of the autonomic nervous system, where cold shock activates the sympathetic system (tachycardia) and diving response activates the parasympathetic system (bradycardia). The resulting arrhythmias can lead to incapacitation, inability to swim and drowning, even if the arrhythmia itself is not fatal (4, 5, 12, 13).

The cardiorespiratory and cardiovascular responses to cold water immersion were already shown in 1989 by Tipton with data generated in adult population. After immersion in cold water in the first minute the heart rate increases from 96 beats/minute to 156 beats/minute and the respiratory rate increases from 12 breaths/minute to 66 breaths/minute (14). Tipton et al. also postulates that the respiratory frequency response, as an indicator of respiratory drive, has a peak at water temperatures around 10–15 °C. The rate of skin temperature cooling in 20 s in adults was 0.42 °C/s at 15 °C, 0.56 °C/s at 10 °C, and 0.68 °C/s at 5 °C water temperature, suggesting that an average rate of cooling in skin temperature between 0.42 °C/s and 0.56 °C/s in the first 20 s is sufficient to trigger a maximal respiratory response due to cold shock (5, 9). In 2015, Bird et al. published two studies on the cold shock response in a small group of children (8, 15). After 1 min in 15 °C water, heart rate increased by 26% and respiratory rate by 55%, peaking at 30 s before declining. Compared to adults, children showed a less pronounced response, possibly due to higher oxygen uptake and metabolic heat production (8). A separate study on cold-water-acclimatized children found no changes in cooling rates or initial heart/respiratory responses, but oxygen uptake was lower, and they felt more comfortable. Similar trends were observed in adults, suggesting acclimatization slows cooling rates in both groups (15).

While most studies on the physiology of drowning and cold shock, with the exception of the two studies by Bird et al. (8, 15), have been conducted in adults, there is almost no data on the physiology of swimming and diving in healthy children and adolescents. Due to this gap and the importance of drowning in this population, our research group has already conducted several studies on swimming and diving physiology. After publishing the first real-life data on the diving response in healthy children, which showed a markedly smaller decrease in heart rate compared to adults (16) and a study investigating the adaptation to physical exercise during submersion and apnea in adolescents and young adults (17), we expanded research towards a group of children with chronic diseases or heart defects. While these patients are believed to have limited ability to adapt to immersion and submersion, posing potential risks during aquatic activities, current recommendations are unclear, and sometimes lead to restrictive patient counseling. In this context, our working group conducted two pilot studies on immersion and submersion in young adults with congenital heart defects (18–20). Yet, comparative data from healthy children is still uncomplete but necessary to evaluate the effects of immersion and submersion in children with pre-existing heart conditions. The less pronounced diving response observed in our previous study (16), together with the weaker cold shock reflex reported by Bird et al. (8), suggests a markedly different physiology of swimming and diving, and consequently of drowning, in children compared to adults. The current study aims to investigate the cold shock response during the immersion of healthy children in cold water with greater accuracy by also comparing reactions during cold water immersion and warm water immersion, with the goal of closing the data gap and providing reliable comparative data for future studies in children with pre-existing heart conditions, to ensure safe water activities for this population.

## Methods

### Participants

Healthy voluntary participants were recruited through personal contacts, local surveys and announcements in schools and swimming clubs in the weeks prior to the experiment. Inclusion criteria were age of 8–14 years, ability to swim and overall health, especially no restrictions of cardiopulmonary function. Exclusion criteria were signs of reduced general condition and acute illness, signs of limitations of cardiopulmonary function, e.g., a newly diagnosed heart murmur, and intellectual disability or genetic disease. The participants and their legal guardians gave their informed consent. The study received ethical approval by the ethics committee of University of Leipzig and is listed under the reference 233/24-ek.

### Experimental protocol

Anthropometric data were measured. Data on potential medical history and medication were obtained from personal interviews. The testing took place in two pools of the first division soccer club RB Leipzig e.V. in the Red Bull stadium in Leipzig. The pools were the same size with a depth of 1 m and two steps, and were located right next to each other in the same room. Ambient temperature was around 25 °C, water temperature was 34 °C (close to thermoneutral) in the warm pool and 11 °C in the cold pool. A water temperature of 11 °C was selected, as previous studies in adults demonstrated the cold shock response to be most pronounced at 10–15 °C (5, 9), thereby ensuring comparability with published research. The lower bound of this range was chosen to elicit the strongest possible response, particularly since a previous study in children reported a less pronounced cold shock response compared to adults (8). For comparison, a temperature of 34 °C was used, as it is close to thermoneutral and remains well above the threshold (∼25 °C) at which the cold shock response should be initiated (5, 9). The tests were supervised by two medical doctors, a pediatric nurse and a study nurse.

Prior to the start of the testing, all participants were instructed to the following protocol. The protocol consisted of two parts, one conducted in a warm pool, the other one in a cold pool. The participants wearing swimsuits or shorts quickly entered the warm water with the water reaching up to their necks. After the monitoring outside the pool, including drying and being wrapped in a towel, they were quickly lowered into the cold water in a seated position without moving themselves, again with the water reaching up to their necks. Immersion in both warm and cold water was completed within a maximum of 5 s for all participants.

Protocol:

1. 2 min of monitoring in a sitting position outside the pool
2. 1 min of immersion until the neck in warm (close to thermoneutral) water
3. 2 min of monitoring in a sitting position outside the pool
4. 1 min of immersion until the neck in cold water
5. 2 min of monitoring in a sitting position outside the pool

Transcutaneous oxygen saturation, heart rate and perfusion index (PI), as a measure of peripheral vasoconstriction (21), were recorded continuously by a peripheral pulse oximetry sensor. Respiratory rate was recorded continuously by acoustic monitoring on the throat. These measurements were done using a Masimo Rad-97^TM^ patient monitor with Masimo RD SET neo CS-3 sensors and RAS-45 rainbow acoustic sensors (Masimo Corporation, Irvine, USA).

ECG was conducted continuously. Skin temperature was measured continuously under water (on the participant's hand) as well as above water (on the participant's forehead). ECG and skin temperature measurement were done using a Dräger Infinity® 540 patient monitor (Dräger Medical GmbH, Lübeck, Germany).

### Statistical analysis

For the statistical analysis, IBM SPSS Statistics for Mac (V29) was used. Non-parametric tests were used due to the small sample size and non-normal data distribution. Anthropometric data were analyzed and gender-specific differences were compared by conducting a Mann–Whitney *U*-test. Heart rate and respiratory rate parameters, changes in skin temperature, increases in heart rate and respiratory rate were compared between warm and cold water by using a Wilcoxon signed rank test for dependent samples. The *p*-value is included in the manuscript. The significance level was set at *α* = 0.05.

## Results

In this study, 12 healthy children aged 9–13 years were enrolled, including 4 girls and 8 boys. Table 1 shows the participants characteristics including anthropometric data. No significant differences between girls and boys could be observed.

**Table 1:** Characteristics of the study population.

Subjects characteristics	Total (*n* = 12) mean (min; max)	Female (*n* = 4; 33,3%) mean (min; max)	Male (*n* = 8; 66,6%) mean (min; max)	*p*-value
Age (years)	11 (9; 13)	11 (9; 13)	11 (9; 13)	0.933
Height (cm)	149.1 (134; 164)	154.5 (146; 164)	146.4 (134; 162)	0.214
Weight (kg)	37.2 (26.7; 52.0)	42.3 (34.2; 52.0)	34.7 (26.7; 46.8)	0.214
BMI (kg/m^2^)	16.6 (14.3; 19.7)	17.5 (16.0; 19.3)	16.1 (14.3; 19.7)	0.214

All 12 participants finished the protocol including immersion into warm water (34 °C) and cold water (11 °C). No participant reported symptoms such as discomfort, dizziness, nausea, shortness of breath, or heart palpitations. No adverse events were seen.

The peripherally measured oxygen saturation remained consistently stable, with average values around 97.4%. The perfusion index showed no consistent trend during immersion and was subject to significant fluctuations and inaccuracies due to cold water and low skin temperature.

**Figure 1:**
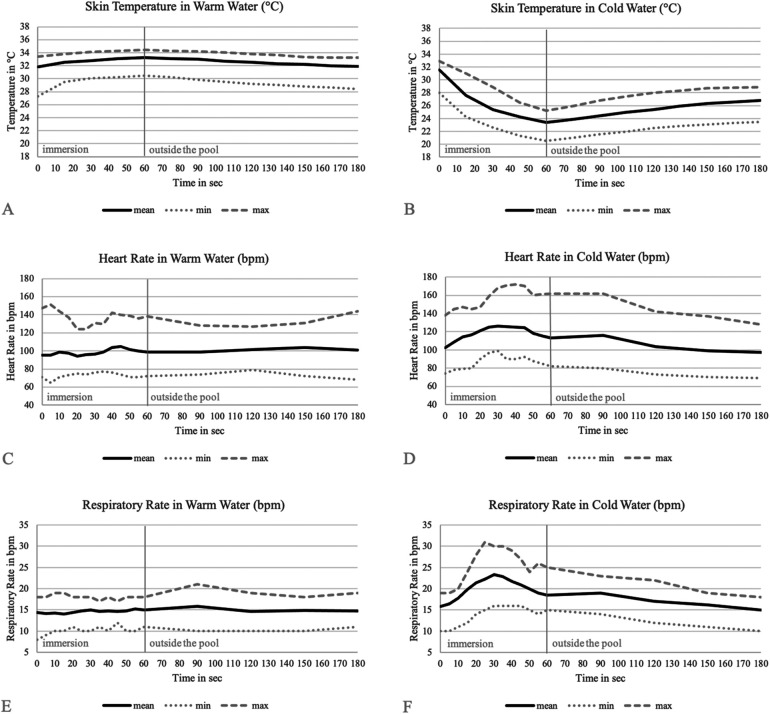
Skin temperature **(A,B)**, heart rate **(C,D)** and respiratory rate **(E,F)** during 60 s of immersion (seconds 0-60) in warm and cold water and 120 s of monitoring outside the pool (seconds 61-180).

In Figure 1, the temporal progressions of hand skin temperature (underwater), heart rate and respiratory rate in warm and cold water are shown. Each graph illustrates 60 s of immersion up to the neck, followed by a 120 s observation period outside the water. Table 2 displays the changes in hand skin temperature, heart rate and respiratory rate over the duration of immersion in warm and cold water.

**Table 2:** Deviation of skin temperature, heart rate and respiratory rate from baseline to maximum in warm and cold water.

Parameters	Warm water mean (min; max)	Cold water mean (min; max)	*p*-value
Changes in skin temperature ( °C)	1.39 (0.6; 3.3)	−8.16 (−7.1; −9.9)	0.002*
Changes in skin temperature (%)	4.55 (1.8; 11.7)	−25.81 (−22; −30.1)	0.002*
Changes in heart rate (bpm)	15.83 (2; 31)	29.5 (15; 51)	0.004*
Changes in heart rate (%)	18.08 (1.6; 38.3)	30.84 (12; 55)	0.005*
Changes in resp. rate (bpm)	2.25 (0; 9)	8.5 (2; 17)	0.018*
Changes in resp. rate (%)	21.78 (0; 100)	58.13 (11.1; 130.8)	0.084

* Significant at a level of *α* = 0.05.

### Skin temperature

As seen in Figure 1, the skin temperature remains relatively constant in warm water and during the following monitoring period, averaging 32.6 °C. In cold water, however, the skin temperature drops from an average of 31.6 °C by around 8.2 °C to 23.4 °C. In the 120 s after emersion, it rises again by approximately 3.4 °C to 26.8 °C, indicating that after 2 min outside the water, full recovery of peripheral skin temperature has not yet been achieved. Table 3 shows the warming and cooling rates of skin temperature in the water. Table 4 presents the temperature differences between head and hand skin temperatures after 60 s in the water. It becomes clear that head skin temperatures remain almost identical in warm and cold water, averaging 35.4 °C, and do not seem to be affected by water temperature. The hand skin temperature in warm water is about 6% lower than the head skin temperature, even though the hand is submerged in 34 °C water and the head is in 25 °C air. In cold water, the difference between head and hand skin temperature is significantly greater with a 34% drop.

**Table 3:** Warming and cooling rates of skin temperature underwater.

Time	Warming rate in warm water ( °C/sec) mean (min; max)	Cooling rate in cold water ( °C/sec) mean (min; max)	*p*-value
After 15 s	0.046 (0.007; 0.173)	0.268 (0.09; 0.48)	0.002*
After 30 s	0.034 (0.007; 0.093)	0.207 (0.127; 0.307)	0.002*
After 45 s	0.028 (0.009; 0.067)	0.163 (0.138; 0.216)	0.002*
After 60 s	0.023 (0.01; 0.055)	0.136 (0.118; 0.165)	0.002*

* Significant at a level of *α* = 0.05.

**Table 4:** Skin temperatures and head—hand differences in warm and cold water.

Parameters	Warm water mean (min; max)	Cold water mean (min; max)	*p*-value
Skin temperature of the head (above water) after 60 s ( °C)	35.44 (34.1; 36.3)	35.43 (34.3; 36.2)	0.838
Skin temperature of the hand (underwater) after 60 s ( °C)	33.22 (30.5; 34.4)	23.44 (20.5; 25.1)	0.002*
Absolute difference btw. head and hand skin temperature ( °C)	−2.23 (−0.8; −5)	−11.99 (−10.5; −14.7)	0.002*
Relative difference btw. head and hand skin temperature (%)	−6.25 (−2.3; −14)	−33.86 (−29.5; −41.8)	0.002*

* Significant at a level of *α* = 0.05.

### Heart rate

Figure 1 displays that in warm water and during the following monitoring period, heart rate remains relatively stable, with an average of 99 beats per minute (bpm). In cold water, in turn, there is a significant increase in heart rate from 102 bpm by 31% to 132 bpm (Figure 1, Tables 2, 5). The increase in heart rate from warm to cold water amounts averages 20 bpm (22%) with a maximum of 48 bpm (57%).

**Table 5:** Heart rate and respiratory rate overall (in total, throughout the entire study), in warm and cold water and the comparison between warm and cold water.

Parameters	Total mean (min; max)	Warm water mean (min; max)	Cold water mean (min; max)	*p*-value
Mean heart rate (bpm)	103.42 (78; 135)	98.33 (74; 137)	118.08 (91; 157)	0.004*
Min heart rate (bpm)	76.58 (58; 114)	88.08 (70; 124)	101.75 (74; 138)	0.025*
Max heart rate (bpm)	138.17 (107; 172)	110.92 (82; 151)	131.83 (107; 172)	0.008*
Heart rate range (bpm)	61.58 (44; 90)	22.83 (11; 38)	30.0 (20; 51)	0.050*
Mean resp. rate (bpm)	16.42 (12; 20)	14.58 (11; 17)	19.92 (15; 24)	0.002*
Min resp. rate (bpm)	10.25 (7; 14)	12.25 (8; 16)	15.58 (10; 18)	0.003*
Max resp. rate (bpm)	24.25 (16; 31)	16.67 (14; 19)	24.25 (16; 31)	0.002*
Resp. rate range (bpm)	14.0 (5; 20)	4.42 (2; 9)	8.67 (2; 17)	0.029*

* Significant at a level of *α* = 0.05.

### Respiratory rate

As seen in Figure 1, respiratory rate remains on a constant level during immersion in warm water and the monitoring period, with an average of 15 breaths per minute (bpm). In cold water, again, respiratory rate significantly increases from 16 bpm by 58% to 24 bpm (Figure 1, Tables 2, 5). The rise of respiratory rate from warm to cold water counts approximately 5 bpm (38%) with a maximum of 10 bpm (91%).

## Discussion

The current study presents new data on the cold shock response in healthy children and the first comparison of physiological reactions during cold water and warm water immersion in this population.

Immersion in warm (close to thermoneutral) water showed no substantial changes in respiratory or heart rate. This is in line with the assumption that a significant change in heart or respiratory rate was not to be expected as neither the cold shock response with tachycardia and hyperventilation nor the diving reflex with bradycardia, triggered by facial submersion or apnea, were present (4). Immersion in water leads to centralization of blood flow to better supply oxygen-dependent organs due to peripheral vasoconstriction and hydrostatic pressure (20, 22), which was demonstrated by the lower hand skin temperature compared to head skin temperature. Thus, the observed immersion responses in children in thermoneutral water are consistent with those previously described in adults. The expected brief increase in heart rate, triggered by the blood shift to central body regions due to hydrostatic pressure during immersion, was not observed. This absence may be explained by a centralization of blood that was either not sufficiently pronounced or not rapid enough to elicit a measurable effect. Furthermore, even before entering the water, a slight increase in heart rate was observed, which may be attributed to excitement or nervousness (arousal).

As described in the literature for adults, the cold shock response and the increase in respiratory rate peak at water temperatures of around 10–15 °C, with the cold shock response lasting for 2–3 min and peaking at 30 s (5, 9, 14). Therefore, assuming the same reactions occur in children, we should observe similar responses to cold water in our experimental setup with a temperature of 11 °C in the cold pool and an immersion time of 1 min. However, it has already been shown that the cold shock response in children is less pronounced, with the data being obtained at slightly higher water temperatures than in the present study (8, 15).

The mean increase in heart rate in cold water is reported to be 62.5% in adults (14) and 26% in children (8). In our pediatric cohort, the heart rate rises during cold water immersion by an average of 31%. When comparing heart rates between warm and cold water, the heart rate in cold water was on average 22% higher than in warm water. Thus, the average heart rate increase in our cohort was lower than that observed in adults, but similar to that previously observed in children, amounting to only one-third or a-half of the increase in adults. However, considering the maximum values with a heart rate increase of 55% during cold water immersion or a 57% higher heart rate in cold water compared to warm water, our results are close to the mean increases reported in adults. In contrast, the differences in the increase in respiratory rate are even more pronounced. In adults, the average increase in respiratory rate in cold water is reported to be 450% (14), in children the increase was described as 55% (8). In our subjects, the average increase in respiratory rate during immersion in cold water was 58%, with a maximum of 131%, which is quite similar to what was previously observed in children. In addition, the respiratory rate in cold water was 38% higher than in warm water, with a maximum increase of 91%. These values do not come close at all to the respiratory rate increases observed in adults during cold water immersion.

Maximum increases in respiratory rate were observed at skin temperature cooling rates of 0.42–0.56 °C/sec after 20 s (5, 9). In our studied children, the average cooling rate was 0.27 °C after 15 s, with a maximum of 0.48 °C, and 0.21 °C after 30 s, with a maximum of 0.31 °C. Thus, the cooling rates and skin temperatures potentially required for a maximal response were not achieved. However, it is important to note that skin temperature was measured peripherally on the back of the hand, which was already significantly lower than central temperatures before immersion in cold water. It can be assumed that the children were already slightly cooled during the monitoring period prior to entering the water, despite rewarming efforts, resulting in primarily lower peripheral skin temperatures. During the monitoring period outside the water, evaporative cooling may have occurred despite drying, resulting in additional heat loss and skin cooling. This is supported by the observation that, due to the typical reflex peripheral vasoconstriction and central blood redistribution during immersion, hand skin temperature was lower than head skin temperature even in warm water, where the head was exposed to 25 °C air temperature and the hand to 34 °C water temperature. Had skin temperature been measured more centrally, such as on the abdomen, the cooling rates might have been significantly higher.

Nevertheless, the water temperatures were sufficiently cold and the immersion time was long enough to potentially observe a maximal cold shock reaction. In fact, the immersion time was even sufficient to observe a beginning of acclimatization to the cold water in most children. The peak of the cold shock response including heart rate and respiratory rate was observed after approximately 30 s with the beginning of normalization of heart and respiratory rate values after 60 s. In our participant group, there were significant increases in heart and respiratory rates in cold water, along with significant differences between warm and cold water. However, the dramatic increases seen in adults were not observed, suggesting that the cold shock response may be less pronounced in children compared to adults.

As a possible reason for the less pronounced reaction in children, the differing expectations between adults and children regarding cold water could be discussed. Since the response is mediated by the autonomic nervous system, subjective expectations may play a role. In adults, it has been shown that an anxious expectation can increase both the magnitude and duration of the cold shock response (23). Children at this age might not accurately assess the actual coldness when told the water temperature is 11 °C, leading to different expectations. The findings of Bird et al. indicate no significant correlation between cooling rate and perceived thermal sensation or comfort, suggesting that the children in their study may have had limited awareness of their own thermal state (8). This highlights the potential dissociation between physiological cooling and subjective thermal perception in pediatric populations. Additionally, variations in the response of cold receptors or differences in how children perceive coldness could also account for the weaker reaction. However, the most significant factor is likely the baseline skin temperature. In the experiments conducted by Tipton et al. (14), participants were dry and usually dressed, suggesting normal skin temperatures. In contrast, the children examined here were in swimwear and wet, only wrapped in towels during the monitoring period, which means they were slightly cooled beforehand. This could explain the relatively high baseline heart rate observed in some children, also likely reflecting their anxiety and excitement about participating in the experiment.

## Conclusion

The current study presents new data on the cold shock response in healthy children and the first comparison between warm (close to thermoneutral) water and cold water immersion in this population. The results showed that immersion into 11 °C (52 °F) cold water, opposed to immersion in warm water, leads to significant increases in heart rate and respiratory rate. Nevertheless, the cold shock response observed in children was less pronounced than described in adults.

## Limitations

The current study is limited by the small number of participants, the narrow age range of the participants and the male dominance in the gender ratio, therefore a bias due to proband selection is possible. With a small sample size and non-normally distributed data, the statistical power is limited. Furthermore, the measurements were conducted using materials and devices not qualified for use underwater. Although the devices were well-adapted to be waterproof, we cannot completely exclude aberrant values because of off-label use.

## Data Availability

The raw data supporting the conclusions of this article will be made available by the authors, without undue reservation.
